# Thylakoid Nanoarchitecture and Photosystem II Energy Partitioning in Response to Treatment with Huminatrin Concentrate “Boron”

**DOI:** 10.3390/ijms27219664

**Published:** 2026-10-28

**Authors:** Eugene Romanovich Bukhanov, Nikolay Alexandrovich Gaevsky, Olga Alexandrovna Poznakhareva, Alexander Apollinaryevich Tikhomirov, Sofya Avrumovna Ushakova, Vasily Filippovich Shabanov

**Affiliations:** 1 Kirensky Institute of Physics FRC «KSC SB RAS», Academgorodok Str. 50/12, Krasnoyarsk 660036, Russia; 2 Federal Research Center «KSC of SB RAS», Academgorodok Str. 50, Krasnoyarsk 660036, Russia; 3 School of Fundamental Biology and Biotechnology, Siberian Federal University, 79, Svobodny Str., Krasnoyarsk 660041, Russia; 4 Krasnoyarsk Agricultural Research Institute, FRC «KSC SB RAS», Prosp. Svobodnyi, 66, Krasnoyarsk 660041, Russia; 5 Institute of Biophysics of the Siberian Branch of the Russian Academy of Sciences, Krasnoyarsk 660036, Russia

**Keywords:** *Brassica napus*, biostimulant, exogenous treatment, thylakoid membrane, grana morphology, PAM fluorometry, quantum yield, photosynthetic efficiency

## Abstract

Photosynthetic acclimation involves coordinated changes in the structural organization and functional activity of the photosynthetic apparatus. However, the structural basis underlying enhanced photosynthetic activity following biostimulant treatment under non-stress conditions remains insufficiently understood. In the present study, we investigated the effects of exogenous application of the humic-boron fertilizer Huminatrin Concentrate “Boron” on the structural and functional organization of the photosynthetic apparatus in spring rapeseed (*Brassica napus* L.) cv. Siberian. Thylakoid and granum architecture was characterized using transmission electron microscopy and quantitative morphometric analysis, whereas photosynthetic function was assessed using pulse-amplitude-modulated (PAM) chlorophyll fluorescence. Huminatrin treatment was associated with changes in granum organization, including a reduction in the stacking repeat distance (SRD), as well as increases in the apparent electron transport rate (ETR) and the effective quantum yield of photosystem II (PSII) photochemistry (Y(II)). Redistribution of PSII excitation energy was accompanied by a decrease in the regulatory quantum yield in PSII (Y(NPQ)) and an increase in the non-regulated energy loss in PSII (Y(NO)). These changes occurred without evidence of pronounced PSII photoinhibition, indicating that the observed changes in PSII photochemistry were not a response to experimentally induced stress. Structural and functional changes were accompanied by increases in plant and leaf biomass. Taken together, these findings indicate that leaf biostimulation is associated with coordinated remodeling of thylakoid architecture and PSII excitation-energy partitioning under the experimental conditions studied.

## 1. Introduction

The development of precision agriculture aimed at identifying optimal conditions for plant growth provides new opportunities for understanding the micromechanisms of photosynthesis through controlled manipulation of agroecological factors and nutrient management. Modern agricultural systems provide an important framework for investigating the processes and mechanisms underlying strategies for improving photosynthetic efficiency.

However, photosynthetic efficiency is determined not only by the availability of external resources. Plants continuously adjust the organization and efficiency of their photosynthetic apparatus in response to changes in environmental conditions and nutrient supply. This acclimation involves coordinated changes at multiple levels, ranging from the composition of light-harvesting pigments and protein complexes to the ultrastructural organization of chloroplasts [1,2,3]. Therefore, understanding the structural basis of processes occurring within the leaf is important for establishing mechanistic links between specific treatments and regulatory changes in the photosynthetic apparatus [2,3].

The thylakoid membrane represents a central structural component of this highly organized system. Within chloroplasts, thylakoid membranes are organized into tightly stacked grana and unstacked stroma lamellae, which provide the structural basis for the organization and interaction of photosynthetic protein complexes [4,5].

Changes in thylakoid stacking, the distance between membranes, and granum architecture may affect excitation energy transfer, photochemical charge separation, and the balance between photochemical utilization and non-photochemical dissipation of absorbed light energy [2,5,6], as well as light propagation and its interaction with photosynthetic structures [7]. In particular, recent structural studies have shown that changes in the degree of thylakoid stacking can alter the spatial relationship between photosystem I (PSI) and II (PSII) and thereby affect the conditions for electron transfer between the two photosystems [8].

The functional consequences of changes in the photosynthetic apparatus can be assessed using chlorophyll fluorescence measurements in green leaves. In particular, pulse-amplitude-modulated fluorescence measurements provide quantitative parameters describing PSII photochemistry and excitation-energy dissipation [9]. The effective quantum yield of PSII photochemistry (Y(II)), describes the fraction of absorbed excitation energy used for photochemical reactions. The regulated and non-regulated energy dissipation pathways are described by the regulatory quantum yield in PSII (Y(NPQ)) and the non-regulated energy loss in PSII (Y(NO)), respectively. The electron transport rate (ETR) provides additional information on the capacity for photosynthetic electron transport under different structural states [9,10]. Thus, combining these functional optical parameters with chloroplast ultrastructural characteristics enables a more comprehensive description of photosynthetic microprocesses than measurements of structure or fluorescence alone [3,9].

Previous studies have shown that structural changes in thylakoid membranes induced by light, salt, or water stress are correlated with changes in PSII quantum yield and electron transport rate, supporting the use of these approaches for non-invasive assessment of the photosynthetic apparatus [11,12,13].

A substantial proportion of studies investigating the effects of biostimulants has focused on their ability to mitigate pre-existing stress [14,15].

In contrast, considerably fewer studies have addressed changes in the photosynthetic apparatus of plants under non-stress conditions. The structural basis of enhanced photosynthetic efficiency under conditions of minimal stress therefore remains insufficiently understood [16].

At the molecular level, thylakoid architecture is dynamically regulated through reversible modification and rearrangement of photosynthetic protein complexes. Phosphorylation of LHCII through the STN7–PPH1/TAP38 system is associated with excitation redistribution between the photosystems and changes in thylakoid organization, whereas STN8 and PBCP predominantly regulate phosphorylation of PSII core proteins and contribute to PSII organization and repair [17,18]. In parallel, interactions between PSII–LHCII supercomplexes in adjacent membranes contribute to grana appression, while CURT1 proteins regulate membrane curvature and granum architecture [19,20]. These mechanisms provide a molecular basis for translating changes in the organization of pigment–protein complexes into measurable remodeling of thylakoid stacking and photosynthetic function.

Thylakoid architecture may also influence photosynthesis through the physical properties of the membrane system itself. Periodically organized photosynthetic membranes can exhibit photonic effects that modify the propagation and local distribution of light [21,22]. Nanophotonic studies of higher-plant thylakoids have shown that their optical response is sensitive to nanoscale membrane organization and the dielectric geometry of the granum [22]. More generally, both the density of available photonic states and the local electromagnetic field depend on the organization of a structured dielectric medium and can influence optical-transition processes [23,24]. Thus, remodeling of thylakoid organization may modify the local photonic environment of photosynthetic complexes and thereby alter the physical conditions governing their interaction with light.

In the present study, we investigated the effects of the humic-boron fertilizer Huminatrin Concentrate “Boron” on the structural and functional organization of the photosynthetic apparatus in spring rapeseed (*Brassica napus* L.) cv. Siberian. Chloroplast and thylakoid ultrastructure was examined using transmission electron microscopy and quantitative morphometric analysis, while PSII functional state was assessed using pulse-amplitude-modulated (PAM) chlorophyll fluorescence. We investigated treatment-associated changes in granum morphology, thylakoid stacking, ETR, Y(II), Y(NPQ), Y(NO), and the spectral characteristics of chlorophyll fluorescence. By comparing structural and functional parameters, we aimed to determine whether changes in thylakoid organization were accompanied by changes in PSII function and to evaluate the potential of combined structural and optical parameters as indicators of the photosynthetic response to exogenous treatment. In addition, a structural–optical model based on the experimentally determined thylakoid organization was used to evaluate how treatment-associated membrane remodeling affects the photonic density of states, the local electromagnetic field, and the resulting Fermi-type optical coupling.

## 2. Results

### 2.1. Plant and Leaf Biomass

**Figure 1:**
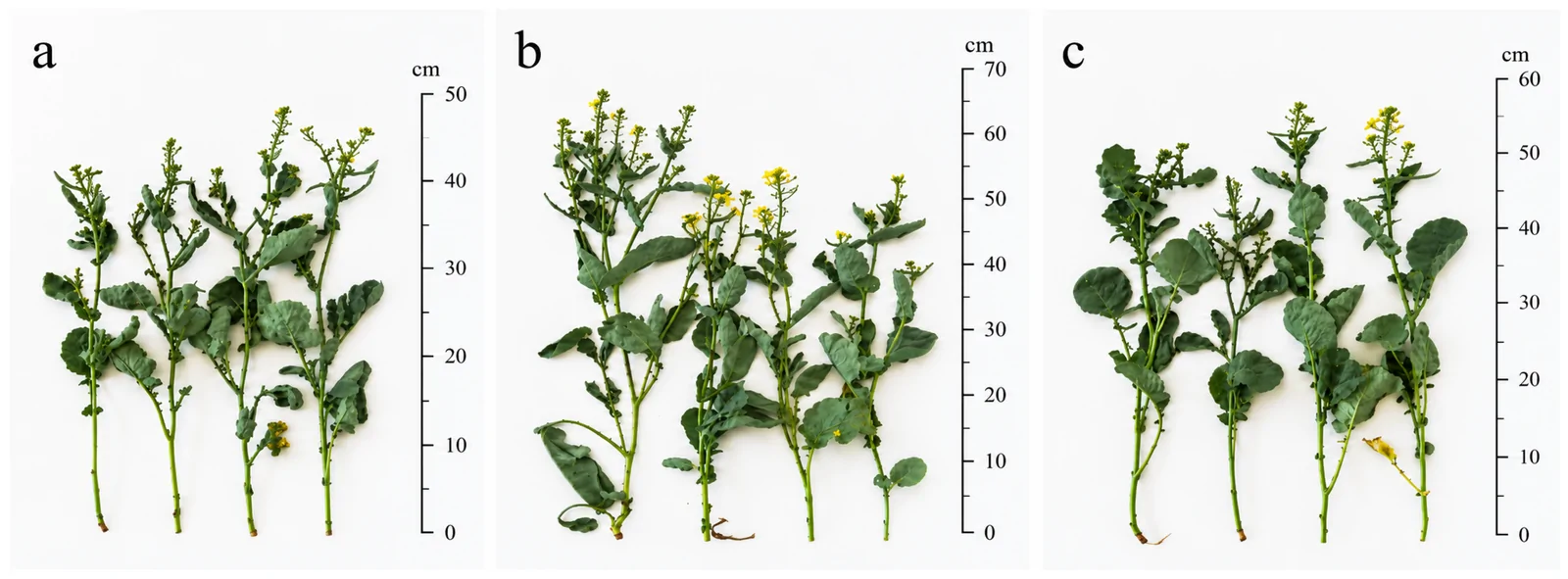
Photographs of plants taken 52 days after emergence. (**a**) Control, without Huminatrin Concentrate “Boron” treatment. (**b**) Variant 1, seeds treated with Huminatrin Concentrate “Boron”. (**c**) Variant 2, seeds treated with Huminatrin Concentrate “Boron” and two applications during vegetation.

Photographs of rapeseed plants taken 52 days after emergence demonstrated visible differences between control and treated plants (Figure 1). Plants in Variant 1 (seed treatment only) and Variant 2 (seed treatment plus two foliar applications to vegetating plants) were characterized by greater height and more developed vegetative biomass compared with the control.

Comparison of plant biomass among treatments at day 52 of vegetation confirmed the visual observations (Table 1). Fresh plant mass in Variants 1 and 2 was 1.6- and 1.5-fold higher, respectively, than in the control, whereas dry mass was 1.5- and 1.4-fold higher, respectively. Fresh leaf mass in Variants 1 and 2 exceeded that of the control by 2.1- and 2.0-fold, respectively, while dry leaf mass was 1.7- and 1.9-fold higher, respectively. Differences between Variants 1 and 2 in both total plant mass and leaf mass were not statistically significant. Thus, treatment with Huminatrin applied to seeds alone resulted in a significant increase in vegetative plant biomass compared with the control. Additional application of the fertilizer during rapeseed development at the stem elongation and budding stages did not significantly affect plant biomass compared with plants grown from Huminatrin-treated seeds alone.

Comparison of fresh and dry mass of the flag leaves in Variant 1 and the control did not reveal significant differences. Fresh and dry mass of the flag leaves in Variant 2 were 1.9- and 1.8-fold higher, respectively, than in the control and 1.6-fold higher than in Variant 1 (Table 2). The flag-leaf area also differed among treatments, averaging 27±2 cm^2^ in the Control, 34±5 cm^2^ in Variant 1, and 19±4 cm^2^ in Variant 2 (Table 2). Thus, the increased flag-leaf mass in Variant 2 was not associated with a larger leaf area.

Application of Huminatrin to vegetating plants resulted in a significant increase in leaf mass per unit area compared with the control and Variant 1 when calculated on a dry-mass basis, by 1.2- and 1.3-fold, respectively. When calculated on a fresh-mass basis, an increase in leaf mass per unit area in Variant 2 was also apparent compared with the other treatments, although the difference was not statistically significant. Seed treatment alone had no significant effect on this parameter compared with the control (Table 2).

Malondialdehyde (MDA) is widely used as a marker of lipid peroxidation intensity, and its accumulation generally increases with the severity of oxidative damage. Therefore, MDA content can be used as an indicator in studies of plant growth dynamics and plant stress responses. In Variant 2, MDA content decreased by 25% and 27% on a fresh- and dry-mass basis, respectively, compared with the control. Variant 1 showed an intermediate level of MDA accumulation and did not differ significantly from either the control or Variant 2, except for MDA content calculated on a dry-mass basis. In Variant 1, MDA content was significantly 29% higher than in Variant 2. Thus, combined seed and foliar treatment was associated with lower thiobarbituric acid (TBA)-reactive MDA equivalents in the flag leaves of Variant 2. These measurements should be interpreted as an indirect estimate of MDA-associated lipid peroxidation rather than as a fully specific measurement of lipid peroxidation intensity.

### 2.2. Photosynthetic Pigments

Treatment with Huminatrin Concentrate “Boron” did not significantly affect the content or ratios of photosynthetic pigments in the flag leaves of Variant 1 compared with the control (Table 3). Pigment contents in Variant 2 were numerically higher than in the Control and Variant 1, but none of these differences was statistically significant. The pigment ratios remained essentially unchanged among treatments (Table 3).

### 2.3. Thylakoid and Granum Ultrastructure

**Figure 2:**
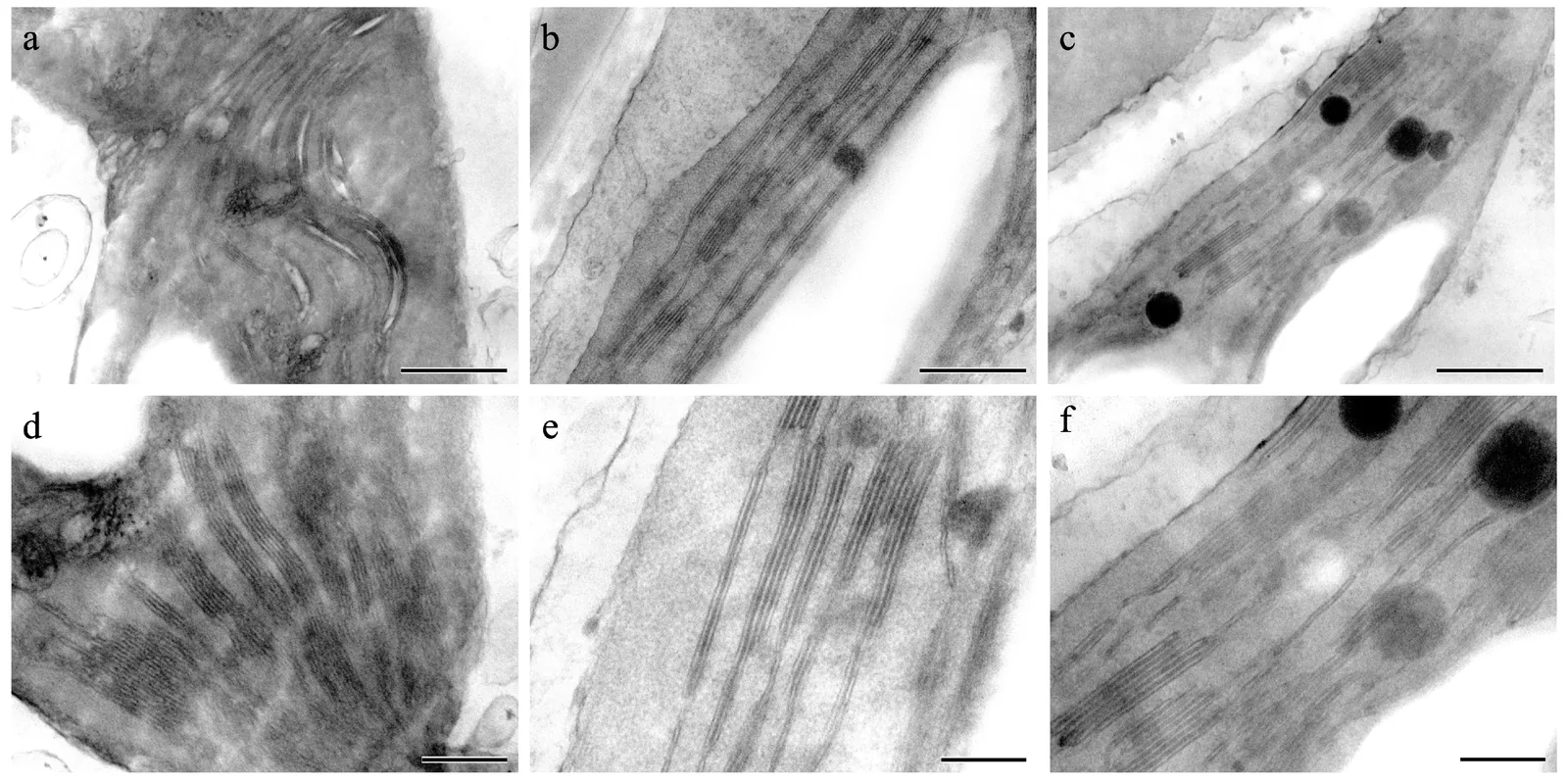
TEM images of rapeseed chloroplast structure for Control (**a**,**d**), Variant 1 (**b**,**e**), and Variant 2 (**c**,**f**) samples. Scale bars: 500 nm (**a**–**c**) and 200 nm (**d**–**f**).

Differences in thylakoid membrane organization between control and treated samples were visually apparent in transmission electron microscopy (TEM) micrographs of chloroplast ultrathin sections (Figure 2). The treated samples showed changes in granum morphology and thylakoid packing compared with the control. In particular, the thylakoid profiles in Variant 2 appeared more clearly defined and compact. However, because visual assessment of individual micrographs does not provide a quantitative estimate of granum dimensions, differences in granum height, diameter, and area were evaluated by morphometric analysis as described below.

**Figure 3:**
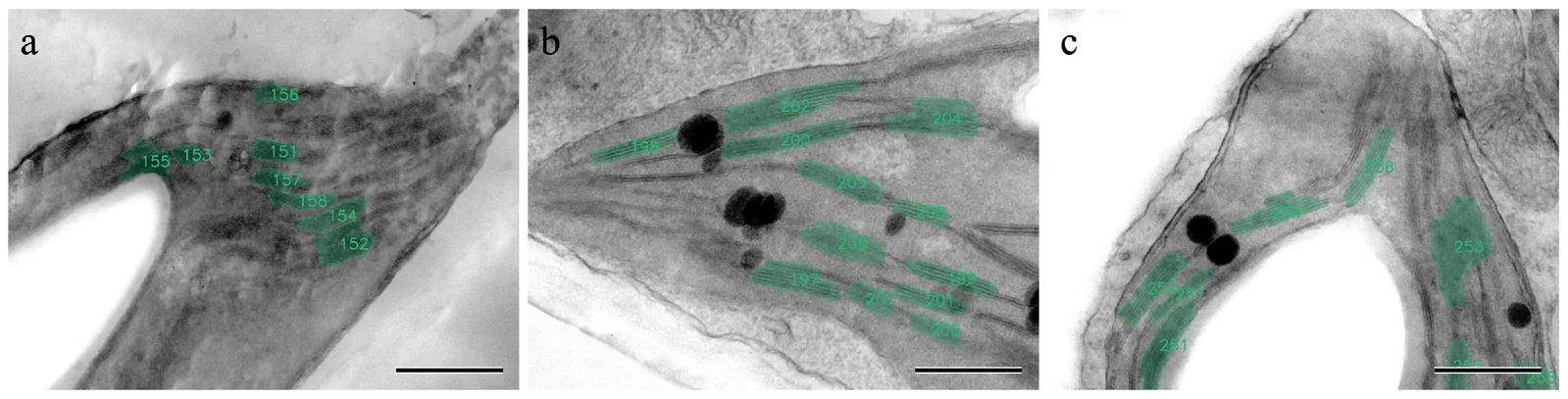
TEM images of rapeseed chloroplast structure for the Control (**a**), Variant 1 (**b**), and Variant 2 (**c**), after processing with the GRANA. Green overlays display software-recognized granum regions. Scale bar: 500 nm.

Visual analysis of the electron micrographs of chloroplasts also revealed substantial differences in the efficiency of automatic granum recognition by the GRANA (Graphical Recognition and Analysis of Nanostructural Assemblies; open-source software, https://github.com/center4ml/GRANA, accessed on 25 October 2026) software among treatments (Figure 3). Although all studied samples underwent sample preparation simultaneously and under identical conditions, it should be noted that the quality of the control samples was much lower than that of the treated samples, which consequently affected the quality of the TEM images. As a result, the number of grana detected using the GRANA software was lower in the control samples than in the treated ones. In control samples, the software recognized approximately 17% of the grana identified by visual inspection of the images. In Variant 1, the proportion of recognized grana increased to 45%, whereas in Variant 2 it reached 68%. Because all samples were subjected to the same sample-preparation procedure, the difference in recognition efficiency was retained as an observation of the image-analysis process. The recognized structures therefore represent treatment-dependent subsets of the visually identifiable granum population.

Automatic analysis of 50 electron micrographs for each experimental treatment revealed changes in the principal structural parameters of chloroplast grana (Table 4). All samples exhibited characteristic values of granum height and the number of thylakoids per stack. The most informative parameter reflecting structural remodeling was stacking repeat distance (SRD). Control samples had the highest SRD values. In Variant 1, this parameter decreased by approximately 7%, indicating tighter thylakoid packing. In Variant 2, SRD had an intermediate value between the control and Variant 1, demonstrating a less pronounced but still noticeable decrease compared with the control.

Violin plots were constructed to visualize the distributions of granum area, perimeter, diameter, and height in the control (Figure S1), Variant 1 (Figure S2), and Variant 2 (Figure S3). The distributions indicate that treatment-associated changes were parameter-specific rather than reflecting a uniform increase in granum size. Granum diameter was shifted toward higher values in both treated variants, with the largest mean diameter observed in Variant 1 (389 nm compared with 297 nm in the control and 328 nm in Variant 2). In contrast, mean granum height remained similar among treatments (128, 124, and 129 nm for the control, Variant 1, and Variant 2, respectively). Granum area and perimeter increased in Variant 1 but were slightly lower in Variant 2 than in the control. Thus, the morphometric distributions support treatment-associated remodeling of granum geometry rather than a uniform enlargement of grana. Together with the lower SRD values in both treated variants, these observations indicate changes in both granum morphology and thylakoid packing following Huminatrin treatment.

### 2.4. Chlorophyll Fluorescence and PSII Function

The dark-adapted fluorescence parameters Fo, Fm, and Fv/Fm did not differ significantly among treatments. In contrast, pronounced treatment-associated differences were observed in the light-adapted parameters describing PSII photochemistry and excitation-energy partitioning, including apparent ETR, Y(II), Y(NPQ), Y(NO), and the Y(II)/Y(NPQ) ratio (Table 5).

Variable fluorescence (Fv/Fm), in contrast, is a relatively stable parameter and ranged from 0.780 to 0.820 in the present study. These values are consistent with a photosynthetic apparatus that was not subjected to pronounced stress.

PAM fluorescence measurements revealed statistically significant differences between the control and treated samples in several energy-related chlorophyll fluorescence parameters, including ETR, Y(II), Y(NPQ), and Y(II)/Y(NPQ) (Table 5).

### 2.5. Optical Modeling of Thylakoid Organization and Fermi-Type Optical Coupling

To examine the optical consequences of the experimentally observed thylakoid remodeling, calculations were performed for structural models based on the measured SRD and mean number of thylakoids per granum. The total granum height was fixed at 127 nm for all treatments. The experimentally determined mean thylakoid numbers of 6.50, 6.94, and 6.88 for the Control, Variant 1, and Variant 2, respectively, were represented by ensembles of grana containing six or seven thylakoids in proportions reproducing the corresponding mean values. This representation resulted in a slightly greater contribution of membrane material within the granum in both treated variants than in the Control.

**Figure 4:**
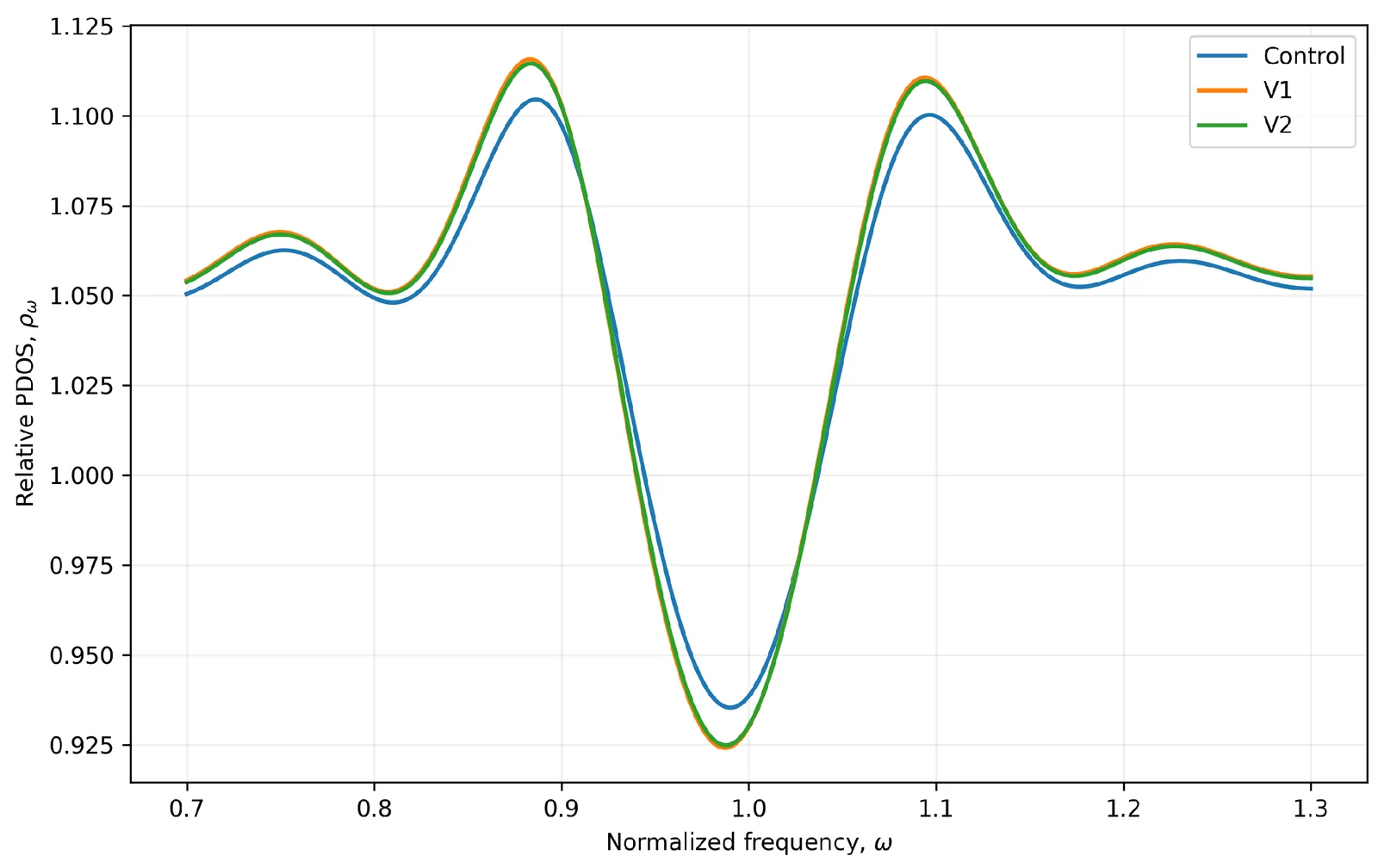
Ensemble-averaged PDOS calculated for granum models corresponding to the Control, Variant 1, and Variant 2 treatments.

The similar optical path lengths of the three structural models resulted in photonic density of states (PDOS) spectra whose principal maximum was located in the same spectral region (Figure 4). The intensity of the principal PDOS feature was slightly higher in both treated variants than in the Control, with the largest increase observed in Variant 1 and a slightly smaller increase in Variant 2.

The membrane-averaged local-field intensity showed the same general response. Both treated variants exhibited a slight increase relative to the Control, with the strongest response in Variant 1. Under identical transition-dipole and orientation conditions, this corresponded to a higher contribution of the squared optical matrix element in the treated structures. The PDOS contribution changed in the same direction.

When the local-field and PDOS contributions were combined within the framework of Fermi’s golden rule, the calculated Fermi-type optical coupling increased in both treated variants. The increase was most pronounced in Variant 1, whereas Variant 2 showed a slightly smaller response.

**Figure 5:**
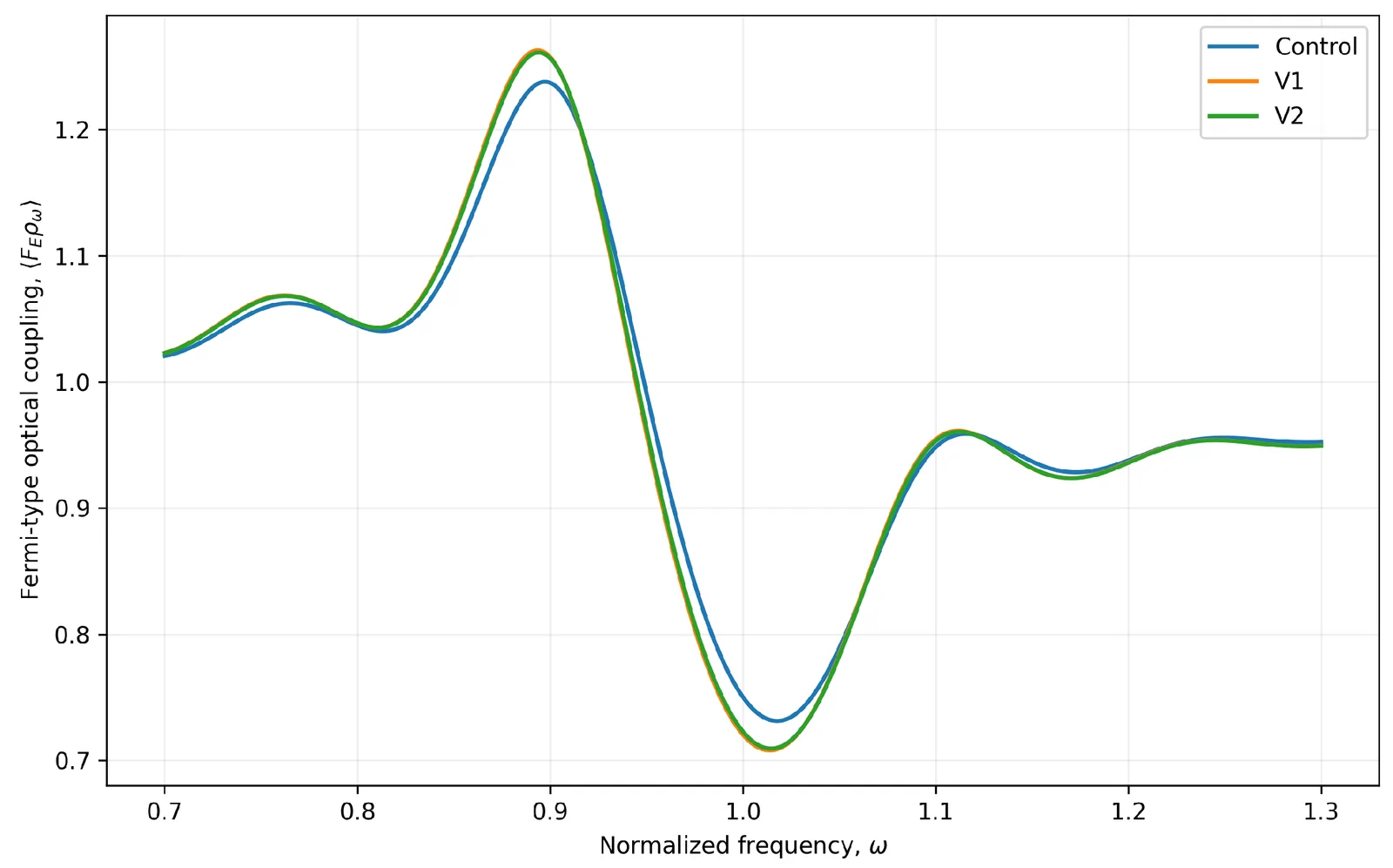
Calculated spectral dependence of the Fermi-type optical coupling, expressed as the ensemble-averaged product $\langle F_{E}\rho_{\omega }\rangle$, for the Control, Variant 1, and Variant 2 treatments.

The spectral dependence of the calculated Fermi-type optical coupling is shown in Figure 5. Both treated variants retained the same general spectral profile as the Control while exhibiting a modest increase in the principal coupling feature.

Overall, the optical calculations showed a consistent treatment-associated increase in PDOS intensity, membrane-localized electromagnetic field intensity, and Fermi-type optical coupling, with the strongest modeled response observed in Variant 1.

## 3. Discussion

The present study demonstrates that treatment with Huminatrin “Boron” was associated with coordinated changes in plant growth, thylakoid ultrastructure, and photosynthetic function. Both treatment regimes resulted in a significant increase in aboveground and leaf biomass compared with the untreated control. At the same time, the two treatment regimes did not differ significantly in total aboveground biomass, indicating that additional foliar applications during plant development did not produce a further significant increase in total plant biomass under the conditions of the present experiment. However, the two treatment regimes affected flag-leaf characteristics differently. Mean flag-leaf area was 27±2, 34±5, and 19±4 cm^2^ in the Control, Variant 1, and Variant 2, respectively; these differences were not statistically significant. Variant 2 nevertheless showed significantly greater flag-leaf mass and dry mass per unit area, whereas Variant 1 had the numerically largest leaf area. These results indicate that the treatment response at the leaf level was not a uniform increase in leaf size but involved treatment-regime-dependent changes in leaf development. The biomass response is consistent with the broader physiological effects expected from a humic-boron treatment, in which changes in photosynthetic activity may occur together with improved nutrient utilization and other growth-related processes.

The obtained results show that Huminatrin application was associated with changes in the structural organization of thylakoid membranes, consistent with previously observed changes in the structural and optical characteristics of rapeseed leaves following exogenous treatment [25].

The functional significance of thylakoid stacking was directly demonstrated by Garty et al. [8], who showed that changes in thylakoid membrane stacking during the dark-to-light transition alter the spatial relationship between PSI and PSII and influence the mode of electron transport. Their experiments showed that specific membrane regions formed during the transition can increase the proximity and connectivity of the two photosystems, thereby reducing spatial constraints on electron transfer between them.

The structural plasticity of thylakoid membranes is also consistent with the broader structural-functional concept proposed by Garab, according to which photosynthetic membranes are dynamically organized supramolecular systems rather than static assemblies [3]. Changes in membrane organization can modify the spatial relationships among photosynthetic complexes and thereby affect light absorption, excitation-energy transfer, and electron transport. Quantitative electron microscopy studies have further demonstrated that thylakoid architecture can be characterized by parameters such as membrane spacing, granum dimensions, and lumen geometry, and that these parameters can change dynamically depending on photosynthetic conditions [26].

The structural response observed here can be considered within the molecular framework established for dynamic thylakoid remodeling. Reversible phosphorylation of LHCII through the STN7–PPH1/TAP38 system regulates excitation redistribution between the photosystems and is associated with changes in thylakoid organization, whereas STN8 and PBCP predominantly regulate phosphorylation of PSII core proteins and contribute to PSII organization and repair [17,18]. In addition, interactions between PSII–LHCII supercomplexes in adjacent membranes contribute to grana appression [19], while CURT1 proteins regulate membrane curvature and granum architecture [20]. Since these molecular mechanisms of thylakoid remodeling have been extensively characterized, the present study addresses a complementary downstream question: how experimentally observed remodeling of thylakoid organization modifies the physical environment of the photosynthetic complexes.

The GRANA analysis revealed treatment-associated differences in granum morphometric parameters, while the efficiency of automatic granum recognition differed among treatments. Therefore, the absolute numbers of software-recognized grana were not interpreted as differences in granum abundance or packing density. Instead, the structural comparison was based on the morphometric characteristics of the recognized granum profiles. These observations are consistent with studies [3,8] demonstrating that granum architecture can change during photosynthetic acclimation.

Structural changes in the thylakoid system were accompanied by pronounced changes in PSII functional parameters. The apparent electron transport rate increased from 106.5±6.4 μmol electrons m^−2^ s^−1^ in the control to 139.8±4.9 and 133.0±3.6 μmol electrons m^−2^ s^−1^ in Variants 1 and 2, respectively. These values correspond to increases of approximately 31% and 25% relative to the control. Importantly, the difference between the two treated variants was not statistically significant. Because the two treated variants did not differ significantly in ETR, additional foliar applications did not result in a further significant increase in apparent electron transport relative to seed treatment alone under the conditions of the present experiment.

As expected from the fluorescence-based calculation of apparent ETR, its increase was accompanied by an increase in Y(II), from approximately 0.40 in the control to 0.50 in both treated variants. The optical calculations provide a quantitative extension of this structural–functional framework. The closely similar optical path lengths of the modeled structures allowed their photonic properties to be considered within the same spectral region. Both treated variants exhibited higher PDOS intensity than the control, with the largest value observed in Variant 1 and a slightly lower value in Variant 2. This behavior is consistent with nanophotonic studies demonstrating that the optical properties of higher-plant grana are sensitive to nanoscale membrane organization [22]. Thus, the experimentally observed remodeling of thylakoid organization can also modify the photonic environment of the membrane system.

The structural basis of this optical response becomes clearer when SRD is considered together with the mean number of thylakoids per granum. The treated variants combined lower SRD values with a higher mean number of thylakoids, resulting in a greater calculated fraction of membrane material within the granum. Because the pigment–protein membrane layers have a higher refractive index than the aqueous compartments, this redistribution of membrane material changes the internal dielectric organization of the granum while maintaining a similar overall optical path. The resulting structural configuration therefore provides a direct connection between the experimentally determined ultrastructure and the calculated optical properties.

Analysis of the local electromagnetic field further showed a slight increase in membrane-averaged field intensity in both treated variants. Under identical transition-dipole and orientation conditions, this corresponded to a higher contribution of the squared optical matrix element, while the PDOS contribution changed in the same direction. When these contributions were combined according to Fermi’s golden rule, the calculated Fermi-type optical coupling was higher in both treated variants, with the strongest response in Variant 1 and a slightly smaller response in Variant 2. Thus, thylakoid remodeling influenced both physical components represented in the model: the local electromagnetic field experienced by the membrane system and the density of available photonic states. The dependence of optical-transition dynamics on the local photonic environment is consistent with established behavior in structured dielectric systems [23].

Taken together, these results support a multilevel interpretation of the treatment response. Molecular regulation provides the established biological basis for dynamic thylakoid remodeling, while the present structural analysis demonstrates treatment-associated changes in SRD and thylakoid organization. The optical calculations show that these structural changes alter the distribution of membrane material within the granum and thereby modify both the local electromagnetic field and PDOS. Their combined contribution increases the calculated Fermi-type optical coupling, whereas PAM fluorometry reveals coordinated changes in PSII excitation-energy partitioning and electron transport. Thus, the structural and optical responses provide complementary levels for describing the functional state of the photosynthetic apparatus.

An important feature of the present experimental design is that the control and both Huminatrin-treated variants were grown simultaneously at the same field site and were exposed to the same natural environmental conditions. No drought, salinity, heat, or other abiotic stress treatment was experimentally imposed on any group. Thus, the term “non-stress conditions” used here does not imply the complete absence of natural environmental fluctuations in the field; rather, it denotes the absence of an experimentally imposed stress differentiating the control and treated plants. The available weather and soil data indicate that the growing season was not characterized by prolonged drought, although transient environmental fluctuations, including periods of high temperature, naturally occurred under field conditions. Importantly, the experimental comparison therefore represents plants developing under the same environmental background rather than a comparison between stressed and unstressed groups. This distinguishes the experimental design from many studies in which biostimulants are primarily evaluated for their ability to mitigate pre-existing stress. In such experiments, improvement of photosynthetic parameters may represent either direct stimulation by the treatment or restoration/preservation of photosynthetic function following stress-induced damage.

Although Variant 1 showed a numerically lower mean Fv/Fm value than the control, this difference was not statistically significant and remained within the observed variability of the measurements. Overall, the Fv/Fm measurements provided no evidence of pronounced PSII photoinhibition in either the control or treated plants.

Carbohydrates, amino acids, phenols, and other constituents of plant extracts can react with TBA, yielding variously colored products that may elevate the optical signal. Nevertheless, this method for assessing the extent of lipid peroxidation is still employed and enables evaluation of the state of cellular membranes, albeit with certain limitations. Biochemical measurements further support the conclusion that treated plants did not experience enhanced membrane damage. TBA-reactive MDA equivalents decreased in Variant 2 compared with the control by approximately 25% when expressed per unit fresh mass and approximately 27% when expressed per unit dry mass. Variant 1 showed an intermediate response. Thus, treatment did not result in increased accumulation of TBA-reactive products; rather, the more intensive treatment regime was associated with lower MDA accumulation. The lower values observed in Variant 2 indicate lower levels of TBA-reactive products under the conditions of the assay. Therefore, the present data do not indicate substantial treatment-associated damage to the photosynthetic apparatus resulting from stress.

This distinction is important for interpreting biostimulant responses. Studies conducted under non-stress conditions have shown that plant biostimulants can enhance photosynthetic activity and growth without acting primarily as stress-mitigation agents. For example, foliar application of protein hydrolysates has been associated with improved photosynthetic activity and growth in plants maintained under non-stress conditions. In a recent study [27], foliar application of animal-derived protein hydrolysates to petunia increased net photosynthesis, stomatal conductance, and fluorescence parameters compared with the control under conditions without additional stress. Similar effects have been reported in tomato [28], where different foliar biostimulant application regimes were compared and the authors reported increases in stomatal conductance and chlorophyll content. Comparable effects have also been described in cucumber [29], where treatment with biostimulants based on vegetable oils and micronutrients increased net photosynthesis, transpiration, ETR, and Fv/Fm and increased fruit mass and length without experimentally imposed stress conditions. These studies indicate that biostimulants may act not only by protecting plants against unfavorable environmental conditions but also by modifying physiological processes in otherwise normally developing plants. The present results extend this concept by providing evidence of concomitant changes at the level of thylakoid membrane architecture.

Thus, the key distinction of the present study is not that Huminatrin produces a positive effect in the complete absence of any environmental stress, but rather that the observed enhancement of photosynthetic function was not associated with recovery from an experimentally induced stress state or with detectable PSII damage. This allows the structural changes in the thylakoid system to be considered as part of a positive physiological response associated with treatment rather than as a response to the recovery of a damaged photosynthetic apparatus.

The results are particularly interesting because the structural and modeled optical responses were accompanied by coordinated functional changes measured by PAM fluorometry. Both treated variants exhibited lower SRD, a higher mean number of thylakoids per granum, increased calculated PDOS and local-field intensity, and higher Fermi-type optical coupling than the control. These structural–optical changes occurred together with higher Y(II), higher apparent ETR, altered Y(NPQ) and Y(NO), and increased biomass. The convergence of ultrastructural, optical-modeling, fluorescence-derived, biochemical, and whole-plant responses supports the interpretation that Huminatrin treatment was associated with coordinated modification of the photosynthetic apparatus across several levels of organization.

Because Y(II), Y(NPQ), and Y(NO) represent complementary excitation-energy pathways, the increase in Y(NO) should not itself be interpreted as evidence of improved photosynthetic performance.

Comparison of the two treatment regimes also indicates that the physiological response was not uniform across treatment intensity or tissue level. Variant 1 already showed higher biomass, Y(II), and apparent ETR, whereas additional foliar applications in Variant 2 did not produce a further significant increase in these parameters. At the same time, Variant 2 showed the lowest MDA-equivalent content and the greatest flag-leaf mass and leaf mass per unit area. Although Variant 2 showed a higher leaf mass per unit area, its apparent ETR was not higher than that of Variant 1. Thus, leaf mass per unit area alone does not explain the differences in fluorescence-derived apparent ETR between the treated variants. The two treatment regimes therefore shared several functional responses but differed in structural and biochemical characteristics, suggesting that the response to Huminatrin depended on developmental stage and tissue level.

Overall, the findings support a model in which Huminatrin treatment is associated with remodeling of thylakoid organization and corresponding changes in the local photonic environment of the membrane system. The calculated increases in PDOS, local electromagnetic field intensity, and Fermi-type optical coupling may contribute to the physical conditions favoring more effective photochemical utilization of excitation energy and electron transport in PSII. In this framework, the optical response represents one component of the coordinated structural and functional reorganization of the photosynthetic apparatus. The accompanying increase in biomass provides a whole-plant correlate of these responses.

## 4. Materials and Methods

### 4.1. Plant Material and Growing Conditions

The study was conducted using spring rapeseed (*Brassica napus* L.) cv. Siberian. The field experiment was carried out in 2025 at the Mikhailovskoye Experimental Production Farm of the East Siberian Department of Forage Production, Krasnoyarsk Research Institute of Agriculture—Branch of the Federal Research Center of the Krasnoyarsk Scientific Center, Siberian Branch of the Russian Academy of Sciences (Krasnoyarsk forest-steppe, Uzhur District).

The experiment was conducted in the Krasnoyarsk forest-steppe zone under natural field conditions. Weather conditions during the 2025 growing season were characterized by temperatures and precipitation close to long-term averages in May, a relatively warm and wet June, temperatures close to the long-term average in July, and excessive precipitation in August. In June, the mean monthly temperature was 17.9 °C (+1.8 °C relative to the long-term average), with occasional daytime temperatures of 35–37 °C and 101.8 mm of precipitation. In July, the mean temperature was 17.7 °C and no drought was observed despite precipitation being 11.3 mm below the long-term average. In August, 151.6 mm of precipitation was recorded, corresponding to approximately 2.4 times the long-term average, with a mean temperature of 15.2 °C. The experimental soil was ordinary heavy-loamy chernozem containing 5.8–7.6% humus, 21.8–27.2 mg/100 g mobile phosphorus (Chirikov method), 13.7–18.0 mg/100 g exchangeable potassium, and 35.0 mg/kg nitrate nitrogen, with a pH of 6.9–7.1. Soil moisture was 29.3%, corresponding to 34.8 mm of available soil water.

Sowing was performed within the optimal agronomic period using an Amazon-6 (Amazononen-werke H. Dreyer SE & Co. KG, Hasbergen, Germany) seeder in a solid-row pattern with a row spacing of 15 cm, a seed placement depth of 2–3 cm, and a seeding rate of 2.5 million viable seeds per hectare. To protect the seeds against pests and soil-borne infections, they were treated with Tabu Neo (JSC Firm “Avgust”, Moscow, Russia) at a rate of 6 L/t.

At the rosette stage, before budding, the crops were treated with a tank mixture of the herbicides Galion (JSC Firm “Avgust”, Russia; 0.3 L/ha) and Quickstep (JSC Firm “Avgust”, Russia; 0.6 L/ha). At the budding stage, the crops were treated with a tank mixture of the insecticide Borey (JSC Firm “Avgust”, Russia; 0.1 L/ha), the herbicide Estok (JSC Firm “Avgust”, Russia; 0.03 kg/ha), and the fungicide ProTeb (Proline, Saint Petersburg, Russia; 0.5 L/ha).

Huminatrin Concentrate “Boron” was supplied by the Research and Production Enterprise “Siberian Humates” (Tomsk, Russia). According to the manufacturer’s specification, a 1% solution of humic compounds contained the following macro- and micronutrients: Cu, 468 mg $L^{-1}$; Zn, 142 mg $L^{-1}$; Co, 49 mg $L^{-1}$; Mg, 42 mg $L^{-1}$; B, 3843 mg $L^{-1}$; K, 6486 mg $L^{-1}$; Mn, 133 mg $L^{-1}$; Fe, 214 mg $L^{-1}$; Mo, 137 mg $L^{-1}$; N, 2300 mg $L^{-1}$; S, 490 mg $L^{-1}$; and mobile P, 15,644 mg $L^{-1}$.

The experiment was established according to the following treatment scheme.

Control—no treatment with Huminatrin. Seeds were treated with Tabu Neo (6 L/t); herbicide treatment was applied at the stem elongation stage before budding; insecticide treatment was applied at the budding stage.

Variant 1—seeds treated with Tabu Neo (6 L/t) plus Huminatrin Concentrate “Boron” (0.5 L/t); herbicide treatment at the stem elongation stage before budding; insecticide treatment at the budding stage. Vegetative plants were not treated with Huminatrin.

Variant 2—seeds treated with Tabu Neo (6 L/t) plus Huminatrin Concentrate “Boron” (0.5 L/t); two foliar applications of Huminatrin Concentrate “Boron” (0.5 L/ha): the first at the stem elongation stage before budding, in a tank mixture with herbicides, and the second at the budding stage, in a tank mixture with the insecticide and fungicide.

Plant samples were collected 52 days after full emergence from the flag leaves (seventh leaf) for all rapeseed treatments.

The physiological state of the photosynthetic apparatus of the flag leaves was assessed using the following parameters: fresh and dry leaf mass, leaf mass per unit area, TBA-reactive MDA equivalents, photosynthetic pigment content, PAM fluorometry and chloroplast ultrastructure.

### 4.2. Determination of TBA-Reactive MDA Equivalents

TBA-reactive MDA equivalents in rapeseed leaves were determined using the thiobarbituric acid colorimetric assay. Optical density was subsequently measured using a UV-2804 spectrophotometer (United Products & Instruments, Int., Dayton, NJ, USA) at 532 nm [30].

### 4.3. Photosynthetic Pigment Analysis

Photosynthetic pigments were extracted from flag leaves using 96% ethanol. A sample of at least 0.4 g fresh mass was used for extraction. The concentrations of chlorophyll a, chlorophyll b, and carotenoids in the extracts were determined using a UV-2804 spectrophotometer (UNICO, Dayton, NJ, USA) at 664, 648, and 439.5 nm, respectively. Chlorophyll concentrations were calculated using the extinction coefficients and equations reported by Wintermans J.E.G. and De Mots A. [31], which were specifically developed for chlorophyll determination in 96% ethanol.

### 4.4. Transmission Electron Microscopy

Chloroplast ultrastructure was examined using TEM. Leaf fragments were fixed in 2.5% glutaraldehyde in cacodylate buffer (pH 7.4), followed by post-fixation in 1% osmium tetroxide. Samples were dehydrated through a graded ethanol series and acetone and subsequently embedded in epoxy resin (Epoxy-Embedding 812 kit, Sigma-Aldrich, St. Louis, MO, USA). All samples were processed in parallel using the same fixation, dehydration, embedding, sectioning, and contrasting procedures. Therefore, differences in image quality were not intentionally introduced by the sample-preparation protocol. Nevertheless, specimen-dependent differences in tissue preservation and ultrastructural contrast may affect the visibility of grana in individual micrographs.

Ultrathin sections 20–30 nm thick were prepared using a Leica EM UC7 ultramicrotome (Leica Microsystems GmbH, Vienna, Austria) and subsequently contrasted with uranyl acetate and lead citrate. Images were acquired using a Hitachi HT7700 transmission electron microscope (Hitachi High-Tech Corporation, Tokyo, Japan) at an accelerating voltage of 90 kV in bright-field mode. For each treatment, 50 electron micrographs were analyzed at a magnification of 15,000×, corresponding to a scale bar of 500 nm.

### 4.5. Morphometric Analysis of Chloroplast Grana

Quantitative analysis of granum morphometric parameters was performed using GRANA, an artificial-intelligence-based tool designed for automatic recognition of grana in electron microscopy images of the thylakoid network and calculation of their structural parameters. The software automatically calculates area, perimeter, diameter, granum height, and the number of thylakoids within a stack. GRANA additionally calculates the SRD and the granum cross-sectional irregularity coefficient (GSI). SRD represents the mean repeat distance between successive thylakoid layers within a granum stack, corresponding to the thylakoid thickness together with the adjacent stromal gap. GSI is a dimensionless measure of granum cross-sectional irregularity ranging from 0 to 1; higher values indicate greater lateral irregularity of the thylakoid stack. GRANA first identifies the granum profile and its orientation and then derives the morphometric parameters from the recognized structure [32]. Curved grana were not excluded solely because of curvature and were included when the stack was sufficiently visible and recognized by the software. A detailed description of the GRANA workflow and parameter calculation is provided in [32].

### 4.6. PAM Fluorometry

Chlorophyll fluorescence was recorded using a LI-6800 system (LI-COR, Inc., Lincoln, NE, USA). Leaves were placed in the fluorometer chamber without detaching them from the plant. Each treatment was measured using four independent replicates. Fluorescence was excited from the adaxial leaf surface on both sides of the midrib in the central region of the leaf blade.

The physiological state of the photosynthetic apparatus was assessed based on chlorophyll a fluorescence parameters obtained during recording of the light-induced fluorescence induction curve (Kautsky curve). The induction curve was recorded for 6 min, with 11 saturating pulses applied at 30 s intervals. Actinic illumination was set to 390 µmol photons $m^{-2}$ $s^{-1}$. The saturating pulse intensity was 15,000 µmol photons $m^{-2}$ $s^{-1}$ for 0.8 s. Prior dark adaptation of the rapeseed plants lasted 20 min. The physiological state of the photosynthetic apparatus was assessed based on the parameters described in [33].

### 4.7. Statistical Analysis

Tables present mean values ± standard errors of the mean. Statistical analysis was performed using Student’s *t*-test to assess the significance of differences between treatment means at a significance level of p≤0.05.

### 4.8. Optical Modeling of the Thylakoid System

#### 4.8.1. Construction of the One-Dimensional Thylakoid Model

To evaluate the optical consequences of the experimentally observed thylakoid organization, the granal system was represented as a finite one-dimensional multilayer structure with light propagating normally to the membrane planes. The model contained eight grana separated by 100-nm aqueous regions. A common granum height of 127 nm, corresponding to the mean of the experimentally determined values for the three treatments, was used in all calculations.

Each thylakoid was represented by a membrane–lumen–membrane unit (M–L–M). The membrane thickness was set to $d_{M}=4.8$ nm [26]. For each treatment, the lumen thickness was calculated from the experimentally determined SRD as

(1) $$d_{L}=\left(D-2d_{M}\right)\times 0.59144,$$

where *D* is the treatment-specific SRD and the coefficient 0.59144 represents the lumen fraction of the non-membrane part of the thylakoid repeat derived from the structural parameters reported for light-adapted thylakoids [26].

The experimentally determined mean numbers of thylakoids per granum were 6.50, 6.94, and 6.88 for the Control, Variant 1, and Variant 2, respectively. Since individual grana contain an integer number of thylakoids, each treatment was represented by granum structures containing either six or seven thylakoids. For a granum containing *N* thylakoids, the stromal separation between adjacent thylakoids was defined by the fixed granum height:

(2) $$d_{S}\left(N\right)=\frac{H-N(2d_{M}+d_{L})}{N-1},\;H=127\;\mathrm{nm}.$$

The sequence within each granum was therefore

(3) $$M-L-M-{\left(S-M-L-M\right)}_{N-1}.$$

The resulting structural parameters are summarized in Table 6.

The refractive index was set to $n_{M}=1.55$ for the pigment–protein membrane layers [22] and to $n_{\mathrm{aq}}=1.33$ for the lumen, stromal and intergranal aqueous regions, and surrounding medium. The same refractive indices were used for all three treatments. The optical path length of each configuration was calculated as

(4) $$L_{\mathrm{opt}}=\sum_{j}n_{j}d_{j}.$$

#### 4.8.2. Statistical Representation of Thylakoid Number

The fractional mean number of thylakoids was represented as a statistical mixture of the two adjacent integer states. The probabilities of six- and seven-thylakoid grana were selected so that their ensemble mean reproduced the experimentally determined value:

(5) $$p_{7}=\bar{N}-6,\;p_{6}=1-p_{7},$$

where $\bar{N}$ is the experimental mean thylakoid number.

For the eight-granum structure, all $2^{8}=256$ possible arrangements of six- and seven-thylakoid grana were calculated. For a configuration containing *k* seven-thylakoid grana, the statistical weight was

(6) $$w_{c}=p_{7}^{k}p_{6}^{\;8-k}.$$

Any calculated optical quantity *X* was then ensemble-averaged as

(7) $$\langle X\rangle =\sum_{c}w_{c}X_{c}.$$

Thus, the experimental mean thylakoid number was incorporated directly into the spatial distribution of membrane and aqueous layers in the optical model.

#### 4.8.3. Transfer-Matrix Calculation and Photonic Density of States

Electromagnetic propagation through each multilayer configuration was calculated using the transfer-matrix method described previously [34]. Within layer *j*, the electric field was represented as

(8) $$E_{j}\left(z\right)=A_{j}e^{-ik_{j}z}+B_{j}e^{ik_{j}z},\;k_{j}=\frac{2\pi \nu }{c}n_{j},$$

where $A_{j}$ and $B_{j}$ are the amplitudes of the forward- and backward-propagating waves, respectively.

For the lossless multilayer system, the relative photonic density of states was calculated as

(9) $$\rho_{\omega }=\frac{T}{L_{\Sigma }n_{0}^{2}}\sum_{j=1}^{N}\left(|A_{j}{|}^{2}+{\left|B_{j}\right|}^{2}\right)d_{j}n_{j}^{2},\;L_{\Sigma }=\sum_{j=1}^{N}d_{j},$$

where *T* is the transmittance and $n_{0}=1.33$ is the refractive index of the surrounding medium. The treatment-specific PDOS spectra were obtained by weighted averaging over all structural configurations.

The spectral coordinate was expressed as the normalized frequency

(10) $$\omega =\frac{2\pi \nu L_{0}}{c},\;L_{0}=100\;\mathrm{nm}.$$

A common normalized frequency corresponding to the principal PDOS maximum shared by the three treatment models was used for comparative calculations of the local field and Fermi-type optical coupling.

#### 4.8.4. Local Electromagnetic Field

The local electric-field intensity was evaluated within the pigment-containing membrane layers. For each structural configuration, the membrane-averaged local-field factor was calculated as

(11) $$F_{E}=\frac{\sum_{j\in M}\int_{0}^{d_{j}}{\left|A_{j}e^{-ik_{j}z}+B_{j}e^{ik_{j}z}\right|}^{2}dz}{{\left|A_{0}\right|}^{2}\sum_{j\in M}d_{j}},$$

where j∈M denotes the membrane layers. The treatment-specific values were obtained by statistical averaging over the structural ensemble.

#### 4.8.5. Fermi-Type Optical Coupling

The relation between the local electromagnetic field, photonic density of states, and optical-transition probability was considered using Fermi’s golden rule:

(12) $$\Gamma_{i\to f}=\frac{2\pi }{\hbar }{\left|M_{fi}\right|}^{2}\rho_{f},\;M_{fi}=-\mu_{fi}\cdot E_{\mathrm{loc}}.$$

For the same transition dipole and orientational distribution, the relative contribution of the squared matrix element is therefore determined by $F_{E}$. The Fermi-type optical coupling function was calculated directly for each structural configuration and subsequently ensemble-averaged:

(13) $$G_{F}\left(\omega \right)=\langle F_{E}\left(\omega \right)\rho_{\omega }\left(\omega \right)\rangle =\sum_{c}w_{c}F_{E,c}\left(\omega \right)\rho_{\omega ,c}\left(\omega \right).$$

Relative treatment differences were evaluated at the common principal spectral feature using the Control as the reference. The full $G_{F}\left(\omega \right)$ spectra were used to characterize the spectral dependence of the calculated Fermi-type optical coupling.

## 5. Conclusions

The present study demonstrates that exogenous application of Huminatrin Concentrate “Boron” was associated with a coordinated multilevel response in spring rapeseed involving plant growth, leaf development, thylakoid ultrastructure, and PSII photochemistry. Both treatment regimes increased aboveground and leaf biomass, while differences in individual leaf characteristics indicated treatment-regime-dependent effects on leaf development.

Transmission electron microscopy combined with quantitative GRANA analysis revealed remodeling of granum geometry and thylakoid organization, with both treated variants showing reduced SRD and a higher mean number of thylakoids per granum. These structural changes coincided with higher Y(II) and apparent electron transport and with pronounced redistribution of PSII excitation energy, characterized by lower Y(NPQ) and higher Y(NO). The increase in the Y(II)/Y(NPQ) ratio further indicated a shift toward photochemical utilization relative to regulated energy dissipation. Pigment ratios remained stable, while Fv/Fm showed no evidence of pronounced PSII photoinhibition.

Optical modeling based on the experimentally determined thylakoid organization showed that the treated variants had a greater membrane contribution within the granum and exhibited higher PDOS and membrane-localized electromagnetic field intensity than the control. When these contributions were combined within the framework of Fermi’s golden rule, the calculated Fermi-type optical coupling increased in both treated variants, with the strongest modeled response observed in Variant 1. These results show that treatment-associated thylakoid remodeling is accompanied by changes in the local photonic environment of the membrane system.

Because all experimental variants developed simultaneously under the same natural field environment without experimentally imposed abiotic stress, the observed response represents modification of an actively functioning photosynthetic apparatus rather than recovery from experimentally induced stress. Taken together, the convergence of plant-level, biochemical, ultrastructural, structural–optical, and fluorescence-derived responses indicates that Huminatrin treatment is associated with coordinated structural and functional reorganization of the photosynthetic apparatus. The calculated increases in PDOS, local electromagnetic field intensity, and Fermi-type optical coupling may contribute to physical conditions favoring more effective photochemical utilization of excitation energy and electron transport in PSII. These findings expand the concept of biostimulant action beyond stress mitigation and show that agricultural treatment can modify thylakoid organization and its associated photonic environment during normal field development of rapeseed.

## Figures and Tables

**Table 1:** Effect of Huminatrin Concentrate “Boron” on the biomass of spring rapeseed cv. Siberian grown under field conditions.

Parameter	Aboveground Biomass			Leaves		
	Control	Variant 1	Variant 2	Control	Variant 1	Variant 2
Fresh mass, g/plant	14.2±2.3 ^a^	23.1±4.2 ^b^	21.7±2.5 ^b^	4.3±0.7 ^a^	9.0±1.3 ^b^	8.4±1.1 ^b^
Dry mass, g/plant	2.6±0.4 ^a^	4.0±0.5 ^b^	3.7±0.4 ^b^	0.7±0.1 ^a^	1.2±0.2 ^b^	1.3±0.1 ^b^

Comparison was performed between columns within the same parameter. Different letters indicate statistically significant differences between means at p≤0.05, $t_{\mathrm{st}}=2.45$ for n=4.

**Table 2:** Effect of Huminatrin Concentrate “Boron” on the physiological characteristics of flag leaves of spring rapeseed cv. Siberian grown under field conditions.

Parameter	Indicator	Control	Variant 1	Variant 2
Leaf mass	Fresh mass, g/leaf	0.81±0.12 ^a^	0.92±0.15 ^a^	1.51±0.34 ^b^
	Dry mass, g/leaf	0.12±0.02 ^a^	0.13±0.02 ^a^	0.21±0.04 ^b^
Leaf mass per unit area	Fresh mass, g/dm^2^	2.88±0.13 ^a^	2.85±0.11 ^a^	3.23±0.17 ^a^
	Dry mass, g/dm^2^	0.42±0.02 ^a^	0.38±0.01 ^a^	0.50±0.03 ^b^
MDA content	fresh leaf mass, nmol/g	28.3±2.6 ^a^	23.5±2.0 ^ab^	21.1±1.0 ^b^
	dry leaf mass, nmol/g	185.2±17.9 ^a^	174.4±13.7 ^a^	135.0±6.7 ^b^
Leaf area	cm^2^/leaf	27±2 ^a^	34±5 ^a^	19±4 ^a^

Comparison was performed between columns within the same parameter. Different letters indicate statistically significant differences between means at p≤0.05, $t_{\mathrm{st}}=2.45$ for n=4.

**Table 3:** Effect of Huminatrin Concentrate “Boron” on photosynthetic pigment content and ratios in flag leaves of spring rapeseed cv. Siberian grown under field conditions.

Parameter	Fresh Mass, mg/g			Leaf Area, mg/dm^2^		
	Control	Variant 1	Variant 2	Control	Variant 1	Variant 2
Chlorophyll *a*	1.27±0.05	1.25±0.06	1.30±0.07	3.67±0.22	3.56±0.25	4.19±0.22
Chlorophyll *b*	0.39±0.01	0.38±0.02	0.40±0.03	1.11±0.07	1.08±0.08	1.28±0.09
Chlorophylls (*a* + *b*)	1.66±0.05	1.63±0.08	1.69±0.10	4.78±0.29	4.63±0.32	5.47±0.38
Chl *a*/Chl *b*	3.26±0.09	3.29±0.11	3.25±0.05	3.26±0.20	3.29±0.23	3.25±0.23
Carotenoids	0.38±0.03	0.37±0.02	0.39±0.02	1.09±0.07	1.07±0.07	1.25±0.09
Chl (*a* + *b*)/carotenoids	4.41±0.21	4.35±0.12	4.36±0.07	4.41±0.26	4.35±0.30	4.36±0.31

No statistically significant differences were detected between treatments within the same parameter and expression basis at p≤0.05.

**Table 4:** Morphometric parameters of chloroplast grana in rapeseed leaves based on automatic analysis of TEM images using the GRANA software.

Parameter	Control	Variant 1	Variant 2
Area, nm^2^	48,157 ± 36,498	50,737 ± 28,315	45,600 ± 24,268
Perimeter, nm	1102±446	1220±395	1098±339
Diameter, nm	297±101	389±118	328±74
Height, nm	128±58	124±49	129±47
Number of thylakoids, *n*	6.50±0.55	6.94±2.65	6.88±3.02
SRD, nm	19.24±5.41	17.89±1.45	18.64±2.56
granum cross-sectional irregularity, coefficient from 0 to 1	0.18±0.07	0.21±0.09	0.21±0.06
GRANA-recognized profiles, *n*	167	391	269

**Table 5:** PAM chlorophyll fluorescence parameters of flag leaves of spring rapeseed cv. Siberian (52 days after emergence, mean ± standard error, *n* = 4).

Treatment	Fo	Fm	Fv/Fm	ETR	Y(II)	Y(NPQ)	Y(NO)	Y(II)/Y(NPQ)
Control	652±21	3606±2	0.82±0.04	107±6	0.40±0.01	0.40±0.02	0.20±0.01	0.9±0.1
Variant 1	742±37	3369±76	0.78±0.03	140±5	0.50±0.01	0.20±0.01	0.30±0.01	2.2±0.1
Variant 2	677±13	3485±28	0.81±0.03	133±4	0.50±0.02	0.20±0.01	0.30±0.01	1.9±0.2
td Control–Variant 1	−2.1	3.1	0.8	−4.1	−4.1	7.5	−3.3	−11.5
td Control–Variant 2	−1.0	4.3	0.2	−3.6	−3.6	5.0	−4.9	−3.9
td Variant 1–Variant 2	1.7	−1.4	−0.7	1.1	1.1	−1.0	0.2	1.3

Values of Student’s *t*-test exceeding the critical value of 2.45 (shown in bold) are considered statistically significant.

**Table 6:** Structural parameters used in the one-dimensional thylakoid models.

Parameter	Control	Variant 1	Variant 2
SRD, nm	19.24	17.89	18.64
Mean thylakoid number per granum	6.50	6.94	6.88
Granum height, nm	127	127	127
Membrane thickness $d_{M}$, nm	4.800	4.800	4.800
Lumen thickness $d_{L}$, nm	5.701	4.903	5.347
Stromal gap, N=6, nm	7.038	7.996	7.464
Stromal gap, N=7, nm	3.315	4.246	3.729
Intergranal region, nm	100	100	100

## Data Availability

The original contributions presented in this study are included in the article. Further inquiries can be directed to the corresponding author.

## Supplementary Materials

The following supporting information can be downloaded at https://www.mdpi.com/article/10.3390/ijms27219664/s1.

## Abbreviations

The following abbreviations are used in this manuscript:

PSII	Photosystem II
PSI	Photosystem I
ETR	Electron Transport Rate
NPQ	Non-Photochemical Quenching
SRD	Stacking Repeat Distance
TEM	Transmission Electron Microscopy
MDA	Malondialdehyde
PAM	Pulse-Amplitude-Modulated (fluorometry)
Y(II)	Effective quantum yield of PSII photochemistry
Y(NPQ)	Quantum yield of regulated non-photochemical quenching
Y(NO)	Quantum yield of non-regulated non-photochemical quenching
Fv/Fm	Maximum quantum yield of PSII
